# Bayesian Estimation of Performance Measures of Cervical Cancer Screening Tests in the Presence of Covariates and Absence of a Gold Standard

**Published:** 2008-02-14

**Authors:** Edson Zangiacomi Martinez, Francisco Louzada-Neto, Sophie Françoise Mauricette Derchain, Jorge Alberto Achcar, Renata Clementino Gontijo, Luis Otávio Zanatta Sarian, Kari Juhani Syrjänen

**Affiliations:** 1 Departamento de Medicina Social, Faculdade de Medicina de Ribeirão Preto, Universidade de São Paulo, Ribeirão Preto, SP, Brazil; 2 Departamento de Estatística, Universidade Federal de São Carlos, São Carlos, SP, Brazil; 3 Departamento de Tocoginecologia, Faculdade de Ciências Médicas, Universidade Estadual de Campinas, Campinas, SP, Brazil; 4 Department of Oncology and Radiotherapy, Turku University Hospital, Turku, Finland

**Keywords:** Bayesian analysis, diagnostic tests, latent variables, cervical cytology, visual inspection with acetic acid, Hybrid Capture II

## Abstract

In this paper we develop a Bayesian analysis to estimate the disease prevalence, the sensitivity and specificity of three cervical cancer screening tests (cervical cytology, visual inspection with acetic acid and Hybrid Capture II) in the presence of a covariate and in the absence of a gold standard. We use Metropolis-Hastings algorithm to obtain the posterior summaries of interest. The estimated prevalence of cervical lesions was 6.4% (a 95% credible interval [95% CI] was 3.9, 9.3). The sensitivity of cervical cytology (with a result of ≥ ASC-US) was 53.6% (95% CI: 42.1, 65.0) compared with 52.9% (95% CI: 43.5, 62.5) for visual inspection with acetic acid and 90.3% (95% CI: 76.2, 98.7) for Hybrid Capture II (with result of >1 relative light units). The specificity of cervical cytology was 97.0% (95% CI: 95.5, 98.4) and the specificities for visual inspection with acetic acid and Hybrid Capture II were 93.0% (95% CI: 91.0, 94.7) and 88.7% (95% CI: 85.9, 91.4), respectively. The Bayesian model with covariates suggests that the sensitivity and the specificity of the visual inspection with acetic acid tend to increase as the age of the women increases.

The Bayesian method proposed here is an useful alternative to estimate measures of performance of diagnostic tests in the presence of covariates and when a gold standard is not available. An advantage of the method is the fact that the number of parameters to be estimated is not limited by the number of observations, as it happens with several frequentist approaches. However, it is important to point out that the Bayesian analysis requires informative priors in order for the parameters to be identifiable. The method can be easily extended for the analysis of other medical data sets.

## 1. Introduction

The sensitivity (*S_e_*) and the specificity (*S_p_*) are the two most common measures of the performance of a diagnostic test, where *S_e_* is the probability of a diseased individual to be correctly identified by the test while *S_p_* is the probability of an individual without the disease (or condition) of interest to be correctly identified by the same test. When the outcomes of the diagnostic test are represented in a continuous scale, a cut-off value should be chosen in order to determine when an individual is classified as positive or negative. Generally, individuals with test outcome larger or at least equal to this fixed cut-off are classified as positive while individuals with test outcomes inferior to this fixed cut-off are classified as negative.

Although the real disease status of the individual could be verified by a procedure generically denominated gold standard, it is common to find situations where a proportion of the sampled individuals cannot be verified on their real disease status. The problem can occur especially when the gold standard is an invasive and/or risky procedure and the definitive verification for apparently healthy individuals is thus neither practical nor ethical. In order to overcome this problem, many studies on the evaluation of the diagnostic test are carried out by considering only verified individuals. However, this approach can lead to measures that are usually biased, leading to studies denominated verification bias or workup bias. Unbiased estimators for *S_e_* and *S_p_* are introduced by Begg and Greenes [1] and Zhou [2].

Another problem appears when all individuals can not be verified by a gold standard. This occurs when there is not a definitive test for detection of the disease or the verification by a gold standard is an impracticable procedure according to its cost, accessibility or risks. In this situation, maximum likelihood estimators are proposed by Hui and Walter [3]. However, these estimators are reasonable only in situations where the number of observations is larger or equal to the number of parameters, which is not our case, as we will see later. Free of this limitation, a Bayesian approach was introduced by Joseph et al. [4]. However, the method of Joseph et al. [4] do not consider the presence of covariates, which are very common on data from diagnostic test studies.

The objective of the present study is to verify the performance measures of cervical cytology, Hybrid Capture II (HC II) and visual inspection with acetic acid (VIA) in the detection of cervical precursor lesions, using a Bayesian statistical method that allows for the estimation of these measures, although part of the sampled women was not verified by a gold standard. We also consider the presence of covariates in our study. Since Bayesian methods are based on incorporation of historical information and expert opinion into the modelling strategy (the called prior information), these elements could be too subjective, with source for other bias. In other words, inadequate prior information can imply in a biased estimator. However, a careful verification of the prior information and a subsequent analysis of its changes in the outcomes can result in reasonable estimates for the tests performance measures.

Thus, the new methodological contribution of the present paper is an extension of the Bayesian method proposed by Joseph et al. [4] for estimating the performance measures of screening tests introducing a vector of covariates. The rest of the paper is organized as follows. In Section 2, we discuss the definition of a gold standard in accuracy studies of cervical cancer screening tests. In Section 3, there is a description of the method of Joseph et al. [4] for estimating *S_e_* and *S_p_* related to two diagnostic tests in the absence of a gold standard. We also introduce in this section the notation used in the paper. In the following, we introduce the methodology for estimating *S_e_* and *S_p_* in the presence of a covariate. The cervical cancer screening data set is described in Section 4. The application of the proposed methodology on the analysis of the data set is presented in Section 5. Concluding remarks are given in Section 6.

## 2. Accuracy of Cervical Cancer Screening Tests

South and Central America have some of the highest incidence rates for cervical carcinoma in the world, ranging from 30/100,000 women to 40/100,000 women, or three to four times the incidence in developed countries [5]. In Brazil, crude estimates of incidence and mortality are given by 19,82/100,000 and 4,49/100,000 women, respectively [6]. Thus, it is strongly justified to analyze the accuracy of different diagnostic tools for cervical carcinoma and their efficacy in screening programs.

In assessing the accuracy of cervical cancer screening tests, it is not straightforward to define an ideal gold standard. In many studies, the gold standard for evaluating the accuracy of screening tests in detecting true positive lesions is histopathology. If biopsies are not obtained, colposcopy is accepted as the final diagnosis. However, colposcopy can give many false negative results when used to discriminate between normal and abnormal tissues (see, for example, Mitchell et al. 1998 and Hopman et al. 1998) [7,8].

The reference test used in these studies, defined by the results of histology or colposcopy, is thus subject to errors and its estimates for sensitivity and specificity can be biased. Another type of bias is evident when only a part of the sampled individuals will have their real disease status confirmed by the biopsy and the remainders are not included in the calculations of the sensitivity and specificity. This occurs principally when only the women with positive result for one or more diagnostic tests (or with positive clinical signals) are submitted to the gold standard and this selection results in an overestimated sensitivity and an underestimated specificity [9].

Despite of the appearance of new methods developed to estimate the sensitivity and specificity of screening tests without a gold standard [10] or in the presence of the verification bias [11], many studies on the accuracy of cervical cancer screening tools present biased results due to the limitations of the proposed gold standard. For example, in a recent meta-analysis of the studies on performance of conventional cervical cytology, McCrory et al. (1999) evaluated 939 studies, where 84 took care of the standards established by the authors to guarantee the quality of the results. Of these 84 studies, only three did not have their results affected by the verification bias [12].

Many studies are introduced in the literature in the absence of a gold standard. For instance, Hui and Walter (1980) derived equations that compute estimates and standard errors of sensitivity, specificity and prevalence, without considering a reference test [3]. Joseph et al. (1995) introduced a Bayesian model using latent variables [4], and Dendukuri and Joseph (2001) extended this method to account for conditional dependence between the diagnostic tests [13]. Other important statistical contributions were provided by Faraone and Tsuang (1994) [14], Qu et al. (1996) [15] and Hadgu and Qu (1998) [16].

## 3. The Bayesian Framework

Considering *k* diagnostic tests, let *T_m_* = 1 if the result of test *m* is positive and *T_m_* = 0 if the result of test *m* is negative, for *m* = 1*,* …*, k*. Let *S_e_m__* and S*_p_m__* be the sensitivity and the specificity of the test *m* respectively and let *g* be an observation of a binary latent variable *G*, introduced in the model aiming to simulate a non-observable gold standard [17]. Denoting the set of the observations and this latent variable for the *i*-th individual by x*_i_^T^* = {*t*_1_, *t*_2_, *t*_3_, *g_i_*}, where *T_m_* is an observation of *T_m_*, we have the joint density function

$$\begin{array}{l} f(x_{i})=p^{g_{i}}{\left(1-p\right)}^{1-g_{i}}\prod_{m=1}^{k}S_{e_{m}}^{t_{m_{i}}g_{i}}\\ {\left(1-S_{e_{m}}\right)}^{(1-t_{m_{i}})g_{i}}S_{p_{m}}^{\left(1-t_{m_{i}})(1-g_{i}\right)}{\left(1-S_{p_{m}}\right)}^{t_{m_{i}}(1-g_{i})} \end{array}$$

assuming that the test outcomes are independents. We have 2*k* + 1 parameter to be estimated, or say, *k* pairs (*S_e_m__*, *S_p_m__*), and the prevalence *p*. The likelihood function *L*(*θ*), where *θ* = (*S_e_m__*, *S_p_m__*, *p m* = 1*,* …*, k*) is the vector of parameters, is given by

(1) $$\begin{array}{l} L(\theta )=p^{\sum_{i=1}^{n}g_{i}}{\left(1-p\right)}^{n-\sum_{i=1}^{n}g_{i}}\prod_{m=1}^{k}S_{e_{m}}^{\sum_{i=1}^{n}t_{m_{i}}g_{i}}\\ {\left(1-S_{e_{m}}\right)}^{\sum_{i=1}^{n}(1-t_{m_{i}})g_{i}}S_{p_{m}}^{\sum_{i=1}^{n}\left(1-t_{m_{i}})(1-g_{i}\right)}\\ {\left(1-S_{p_{m}}\right)}^{\sum_{i=1}^{n}t_{m_{i}}(1-g_{i})}. \end{array}$$

The latent variable *G*, following the Bayes equation, has a Bernoulli distribution, that is,

(2) $$G_{i}∼Bernoulli\left(\frac{p\prod_{m=1}^{k}S_{e_{m}}^{t_{m_{i}}}{\left(1-S_{e_{m}}\right)}^{1-t_{m_{i}}}}{p\prod_{m=1}^{k}S_{e_{m}}^{t_{m_{i}}}{\left(1-S_{e_{m}}\right)}^{1-t_{m_{i}}}+(1-p)\prod_{m=1}^{k}S_{p_{m}}^{1-t_{m_{i}}}{\left(1-S_{p_{m}}\right)}^{t_{m_{i}}}}\right).$$

Considering beta prior densities *Beta*(α*_θ_*, *β_θ_*) for all parameters in *θ*, where α*_θ_* and *β_θ_* generically denotes fixed hyperparameters and combining the likelihood function for *θ* (1) with the prior densities, we use the Gibbs sampling algorithm [18,19] to simulate samples for the posterior distribution for *θ*. These samples are simulated from the full conditional posterior distributions for *p*, *S_e_m__* and *S_p_m__*.

Following Equations (1) and (2) and considering *k* diagnostic tests, the conditional posterior distributions for the components of *θ* needed for the Gibbs sampling algorithm are given by

$$\begin{array}{l} P|X,\alpha_{p},\beta_{p}∼Beta(\sum_{i=1}^{n}g_{i}+\alpha_{p};\\ n-\sum_{i=1}^{n}g_{i}+\beta_{p}), \end{array}$$

$$\begin{array}{l} S_{e_{m}}|X,\alpha_{S_{e_{m}}},\beta_{S_{e_{m}}}∼Beta(\sum_{i=1}^{n}t_{m_{i}}g_{i}+\alpha_{S_{e_{m}}};\\ \sum_{i=1}^{n}(1-t_{m_{i}})g_{i}+\beta_{S_{e_{m}}}),\;\text{and} \end{array}$$

$$\begin{array}{l} S_{p_{m}}|X,\alpha_{S_{p_{m}}},\beta_{S_{p_{m}}}\\ ∼Beta(\sum_{i=1}^{n}(1-t_{m_{i}})(1-g_{i})+\alpha_{S_{p_{m}}};\\ \sum_{i=1}^{n}t_{m_{i}}(1-g_{i})+\beta_{S_{p_{m}}}), \end{array}$$

for *m* = 1*,* …*, k*. This model is analogous to model developed by Joseph et al. (1995) [4].

Let **w***_i_* be a sample observation of **W***_i_* , a vector of *L* covariates. For the sake of simplicity and without lack of generality, we assume that *T_i_* is a random variable (with observation *t_i_*) related to result of only one diagnostic test, with Bernoulli distribution with success probability *p_i_S_e_i__* + (1 − *p_i_*) (1 − *S_p_i__*), *i* = 1*,…n.* In the presence of a vector of covariates, let us assume the logit links for *S_e_i__*, *S_p_i__* and *p_i_,* given by *θ_li_* = exp (∑*_j_*_=0_*^L^β_lj_w_ji_*) [1 + exp (∑*_j_*_=0_*^L^β_lj_w_ji_*)]^−1^, where *l* = 1, 2, 3, *W*_0_*_i_* = 1, *θ*_1_*_i_* = *S_ei_* , *θ*_2_*_i_* = *S_pi_*, *θ*_3_*_i_* = *p_i_* , for *i* = 1, …, *n*. In this way, we have a vector of parameters given by *β* = (*β*_1_, *β*_2_, *β*_3_), where *β*_l_ = (*β_l_*_0_, *β_l_*_1_, …, *β_lL_*), *l* = 1, 2, 3. Assuming prior independence among the parameters, we consider the prior densities for *β_lj_* with normal distribution with fixed hyperparameters *a_lj_* (means) and *b_lj_*^2^ (variances), *l* = 1, 2, 3, *j* = 0, 1, …, *L*. The likelihood function for *β* is given by

$$L(\beta )=\frac{\text{exp}\left[\sum_{j=0}^{L}\beta_{1_{j}}\sum_{i=1}^{n}w_{ji}t_{i}g_{i}+\sum_{j=0}^{L}\beta_{2_{j}}\sum_{i=1}^{n}w_{ji}(1-t_{i})(1-g_{i})+\sum_{j=0}^{L}\beta_{3_{j}}\sum_{i=1}^{n}w_{ji}g_{i}\right]}{\prod_{i=1}^{n}\left\{{\left[1+\text{exp}\left(\sum_{j=0}^{L}\beta_{1_{j}}w_{ji}\right)\right]}^{g_{i}}{\left[1+\text{exp}\left(\sum_{j=0}^{L}\beta_{2_{j}}w_{ji}\right)\right]}^{1-g_{i}}\left[1+\text{exp}\left(\sum_{j=0}^{L}\beta_{3_{j}}w_{ji}\right)\right]\right\}},$$

where *g* is an observation of the latent variable *G*, given by (2). Combining the prior distributions with *L*(*β*), we have the conditional posterior distributions for *β* given by the vector of unknown parameters is now given by

$$\begin{array}{l} \pi \left(\beta_{1_{j}}|\beta_{\left(\beta_{1_{j}}\right)},X,W\right)\propto N\left(a_{1_{j}};b_{1_{j}}^{2}\right)\times \text{exp}\left\{\beta_{1_{j}}\sum_{i=1}^{n}w_{ji}t_{i}g_{i}-\sum_{i=1}^{n}g_{i}\text{ln}\left[1+\text{exp}\left(\sum_{k=0}^{L}\beta_{1_{k}}w_{ki}\right)\right]\right\},\\ \pi \left(\beta_{2j}|\beta_{\left(\beta_{2j}\right)},X,W\right)\propto N\left(a_{2_{j}};b_{2j}^{2}\right)\times \text{exp}\{\beta_{2_{j}}\sum_{i=1}^{n}w_{ji}(1-t_{i})(1-g_{i})\\ -\sum_{i=1}^{n}(1-g_{i})\text{ln}\left[1+\text{exp}\left(\sum_{k=0}^{L}\beta_{2_{k}}w_{ki}\right)\right]\},\text{and}\\ \pi \left(\beta_{3_{j}}|\beta_{\left(\beta_{3_{j}}\right)},X,W\right)\propto N\left(a_{3_{j}};b_{3_{j}}^{2}\right)\times \text{exp}\left\{\beta_{3_{j}}\sum_{i=1}^{n}w_{ji}g_{i}-\sum_{i=1}^{n}\text{ln}\left[1+\text{exp}\left(\sum_{k=0}^{L}\beta_{3_{k}}w_{ki}\right)\right]\right\}. \end{array}$$

where *j* = 0, 1*,* …*, L and β* _(_*_β_*__10_)_ is the vector of all parameters except *β*_10_ (for example). Observe that we should simulate samples for all parameters considering the Metropolis-Hastings algorithm [47] since their conditional distributions are difficult to sample. In each cycle of the algorithm is generated a new value for the latent variable *G* as (2).

In studies of the performance of two or more independent diagnostic tests applied to a selected group of individuals, where none of these tests can be considered the gold standard, a straightforward extension of this model can be used. Considering the three diagnostic tests, cervical cytology, VIA and HCII, the vector of unknown parameters is now given by *β* = (*β*_1_, …, *β*_7_), where *β_l_* = (*β_l_*_0_, *β_l_*_1_, …, *β_lL_*), *l* = 1, …, 7, are vectors of parameters related to the sensitivity and the specificity of each test and the prevalence of cervical lesions. Let *T_m_i__* be a random variable with observation *t_m_i__* related to test *m*, *m* = 1, 2, 3. Using logit link function to relate the vector **W***_i_* of *L* covariates to the screening performance measures, *i* = 1*,* …*n*, the likelihood function for *β* is now given by

$$L(\beta )=\frac{\text{exp}\left[\sum_{i=1}^{3}\sum_{j=0}^{L}\beta_{lj}\sum_{i=1}^{n}w_{ji}t_{l_{i}}g_{i}+\sum_{i=4}^{6}\sum_{j=0}^{L}\beta_{lj}\sum_{i=1}^{n}w_{ji}(1-t_{{\left(l-3\right)}_{i}})(1-g_{i})+\sum_{j=0}^{L}\beta_{7j}\sum_{i=1}^{n}w_{ji}g_{i}\right]}{\prod_{i=1}^{n}\left\{\prod_{l=1}^{3}{\left[1+\text{exp}\left(\sum_{j=0}^{L}\beta_{lj}w_{ji}\right)\right]}^{g_{i}}\prod_{l=4}^{6}{\left[1+\text{exp}\left(\sum_{j=0}^{L}\beta_{lj}w_{ji}\right)\right]}^{1-g_{i}}\left[1+\text{exp}\left(\sum_{j=0}^{L}\beta_{7j}w_{ji}\right)\right]\right\}}.$$

In this expression, the vectors of parameters *β*_1_, *β*_2_ and *β*_3_ are related to the sensitivities of the cervical cytology, VIA and HC II, respectively; *β*_4_, *β*_5_ and *β*_6_ are related to the specificities of the cervical cytology, VIA and HC II, respectively; and the vector *β*_7_ is related to the prevalence of cervical lesions. We consider the prior densities for *β_lj_* with normal distribution with fixed hyperparameters *a_lj_* (means) and *b_lj_*^2^ (variances), *l* = 1*, ..,* 7, *j* = 0, 1*,* …*, L*. Combining the prior distributions with *L*(*β*), we have the conditional posterior distributions for *β* and the Metropolis-Hastings algorithm is used to generate samples from the each parameter.

## 4. Data Set

The data set is from a European Commission funded ongoing study known as the LAMS (Latin American Screening) study, where PAP smear/liquid-based cytology and screening colposcopy were compared with three optional screening tools (visual inspection with acetic acid or Lugol’s iodine and cervicography) and with Hybrid Capture II from conventional samples and from self-samples, in women at different risk for cervical cancer in three Brazilian arms (São Paulo, Campinas and Porto Alegre) and one Argentine arm (Buenos Aires). The study design and baseline data of the LAMS study were presented by Syrjanen et al. (2005) [20]. Partial results from the LAMS study were provided by Sarian et al. (2005) [21].

In the present study, we considered the data from Campinas, one of the three Brazilian arms of the LAMS study. From February to December 2002, 1,195 women were recruited at a basic health unit and from July to December 2002, 221 women were recruited at the University Hospital (Centro de Atenção Integral à Saúde da Mulher-CAISM). Both services are situated in Campinas, a 969,396 inhabitants city in Brazil’s southeast region. Among these 1.416 women, 809 women were eligible for the study related to the sensitivity and specificity of three cervical cancer screening tests (cervical cytology, visual inspection with acetic acid and Hybrid Capture II) in the presence of covariates and in the absence of a gold standard and were willing to participate. Women were eligible if they were between 18 and 60 years of age, if they had been submitted at all three diagnostic methods and if they had intact uterus. Patients previously subjected to treatment for condylomas or with history of current abnormal cytology were excluded. Women who presented with confirmed immunossupression, immunodeficiency or HIV infection, who had sexual intercourse or vaginal medication in the last three days were not included. Informed consent was obtained from all participant women. The study protocol was reviewed and approved by the Committee of Ethics in Research of the Medical Science School of the State University of Campinas.

Cervical cytology was collected after evaluation and treatment for possible infectious processes. Ayre spatulas and cervical brushes were used for these samplings. The samples were stained according to the Papanicolaou method and evaluated using the Bethesda System [21]. Cytology was considered positive if showing cellular atypia, irrespective of their severity. Ecto- and endo-cervical samples were collected for second generation Hybrid Capture (HC II) using sterile endo-cervical brushes supplied by Digene Diagnostics and processed following the instructions of the manufacturer (Digene Diagnostics Inc.). The HC-II is a molecular biological method that tests the presence of the HPV-DNA, through a chemoluminescent reaction. HC-II is commercialized as standard kits and it is based on a reaction of hybridization realized into several sorts of solutions with non-radioactive probes of known ribonucleic acids. Viral load was measured in relative light units (RLU/CO) and HC II results were categorized as negative if <1 RLU/CO and positive otherwise. After the collection of the cervical cytology and HC II, dilute 5 percent acetic acid was applied to the cervix. One minute afterwards, the cervix was illuminated with adapted spotlights (100 Watts) and naked-eye examined for acetowhite areas. The visual appearance was classified according to the Atlas for Unaided Visual Inspection of the Cervix [22] using the categories: normal, atypical, intra-epithelial neoplasia or suggestive of cervical cancer. Normal or atypical results were classified as negative and intra-epithelial neoplasia or suggestive of cervical cancer were classified as positive. More details on the study protocol may be found in Syrjanen et al. (2005) [20].

## 5. Results

First of all, the sensitivity and specificity of cervical cytology (*T*_1_), VIA (*T*_2_) and HC II (*T*_3_) were estimated by a Bayesian approach proposed by Joseph et al. (1995) [4]. This method was developed for the situation where a reference test is not available and it has the assumption that the tests are conditionally independents. Seven parameters were estimated, including the prevalence of pre-neoplasic or neoplasic lesions and the sensitivity and specificity pairs relative to the three diagnostic methods under evaluation. An important feature of the Bayesian approach is the combination of the data obtained by the current sampling scheme with prior information about the parameters of interest. This prior information is quantitatively introduced in the statistical analysis and it can represent the pooled subjective opinions of the experts, or information derived from the published literature. In the present study, we initially defined the prior information from the medical literature, using beta probability distributions.

The prior information about the sensitivity and the specificity of cervical cytology was based on the systematic review of Nanda et al. (2000), who presented sensitivity for atypical squamous cells of undetermined significance (ASC-US) or worse being ranged from 29 percent to 56 percent and specificity from 97 percent from 100 percent [23]. The studies of Belinson et al. (2001) and of the University of Zimbabwe and JHPIEGO Cervical Cancer Project (1999) were used as references for the choice of the prior information about the sensitivity and the specificity for the VIA [25–26]. In these studies, the sensitivity of VIA for at least CIN II was estimated in 55 percent and 64 percent, respectively and the specificity was estimated as 76 percent and 67 percent, respectively. The prior information of the accuracy measures of HC II test was based on the studies of Schiffman et al. (2000) and Wright et al. (2000), who estimated sensitivities by 88.4 percent and 81.3 percent (at 1 RLU cut-off), respectively and specificities by 89.0 percent and 84.5 percent, respectively [27–28].

However, the choice of informative prior distributions based only in a summary of previous studies can be a complex task, since each study has elements of subjectivity, error-proneness and possible potential for bias. Thus, a panel of experts on cervical cancer was asked to provide their best estimate for the sensitivities and specificities of the tests and the prior distributions that summarise the information provided by the literature review corrected by the experts were derived. The assessment of beta distribution priors for each test parameter considered the method presented by Joseph et al. (1995) [4], where the hyperparameters are defined by matching the center of a range of plausible values of sensitivity and sensitivity with the mean of the beta distribution and matching the standard deviation of the beta distribution with one quarter of the total range. We considered a vague prior distribution for the prevalence of precursor cervical lesions (a Beta distribution with hyperparameters 0.5 and 0.5, see [29]) motivated by a little background knowledge about this parameter.

The median age of the 809 women who participated of the study was 34 years. Approximately three quarters of these women lived with a partner (73.0 percent) and one-third had 8 or more years of education (33.3 percent). The majority self-reported to be white (67.2 percent) and 64.3 percent reported not to be a smoker. Half of the women (50.3 percent) reported to have had only one lifetime sexual partner and almost three quarters (72.9 percent) had initiated the sexual life in the teenage. Only 1.5 percent of the women entered in the study with less than one year since her first sexual intercourse and the majority (86.1 percent) reported to have had only one sexual partner during the last 12 months. The percentage of women who are pregnant at the time of the study was around 7.7 percent.

Based on the cases for which cervical cytology was available, 758 (93.7 percent) had normal results, 12 (1.5 percent) had low-grade squamous intraepithelial lesion (LSIL), 4 (0.5 percent) had high-grade squamous intraepithelial lesion (HSIL) and 35 (4.3 percent) were ASC-US. Table 1 shows the results of the three tests for the 809 available cases. For a Bayesian data analysis, the Gibbs sampler algorithm was run for 100,000 cycles, where the first 20,000 were used to assess convergence and the last 80,000 were used for inferences. For each parameter of interest, the arithmetic mean of these 80,000 Gibbs samples is a natural Bayesian estimator. These arithmetic means are showed in Table 2, with the respective 95 percent credible intervals. Table 2 also shows positive and negative predictive values for each diagnostic test, calculated in each cycle of the Gibbs algorithm from the estimated sensitivities and specificities and the prevalence figures (a mathematical approach is presented by Altman and Bland [30]).

The results suggest a low sensitivity for cervical cytology to detect ASC-US or worse (53.6 percent) as well as for VIA (52.9 percent), but indicate a high sensitivity for the HC II (90.3 percent). All screening methods presented relatively high specificities, 97.0 percent for cervical cytology, 93.0 percent for VIA and 88.7 percent for the HC II. As is evident in Table 2, all methods presented very low positive predictive values (PPV) due to the low prevalence of cervical lesions [30]. Although with high sensitivity and specificity, HC II did not present a high PPV (35.3 percent), which is similar to that estimated for VIA. On the other hand, all methods presented high negative predictive values (NPV) (Table 2). The prevalence of precursor lesion was estimated as 6.4 percent and this low prevalence naturally imply few diseased individuals and consequently low PPVs.

Another important result from the Bayesian model is related to the estimates for the expected value of true positives for each combination of the three screening methods, as shown in Table 3. This estimator is a numerical approximation and in this way it may accept decimals. The proportion of the predicted number of positives from the total of sampled individuals is the estimate of PPV. In Table 3, we notice that for 9 women, all three tests reported positive outcomes and the expected PPV is 98.6 percent. On the other hand, when all the tests are negative, it is expected that only 0.2 percent of the women with this outcome will have cervical lesions.

Tables 4 and 5 shows as secondary results the sensitivities and specificities for each combination of the screening tests. Table 4 summarizes the results when the tests are evaluated in serial combination (positive when both tests were positive and negative otherwise) and Table 5 summarizes the results when the tests are evaluated in parallel combination (positive when at least one of the tests was positive and negative otherwise). When the association between two tests is considered, the results of the third test are not considered. In Table 4, the serial combination between cervical cytology and visual inspection has a sensitivity of 78.0 percent, which is higher than the sensitivity of each test individually (53.6 and 52.9 percent respectively, see Table 2). This result suggests an apparent improvement in sensitivity, but at a cost in specificity, when these two tests are used jointly. However, this increase in sensitivity must be interpreted with caution. Franco [31] advised that nominal increase in sensitivity always occurs by chance whenever an adjunct test, as HC II, is used in combination with a conventional test, as Pap cytology, even if the complementary test was totally random with respect to the disease being evaluated.

In a second instance, we introduced in the model the age of the women (*X*) as a continuous covariate. The covariate *W*_1_ is given by (*X* − *x̄*)/10, where *x̄* is the sample mean of *X*. The quotient 10 is only considered for avoiding numerical instability related to large values in exponential functions present in the conditional posterior densities of interest. We also introduced in the model the variable *W*_2_, a dichotomous variable that denotes whether or not the woman is actually pregnant (1 if pregnant and zero otherwise). Firstly, we considered 13 the interaction between *W*_1_ and *W*_2_ in the model. However, all interaction parameters were estimated to be close to zero (ranged from −0.031 to 0.009) and it were excluded from the final model.

**Figure 1:**
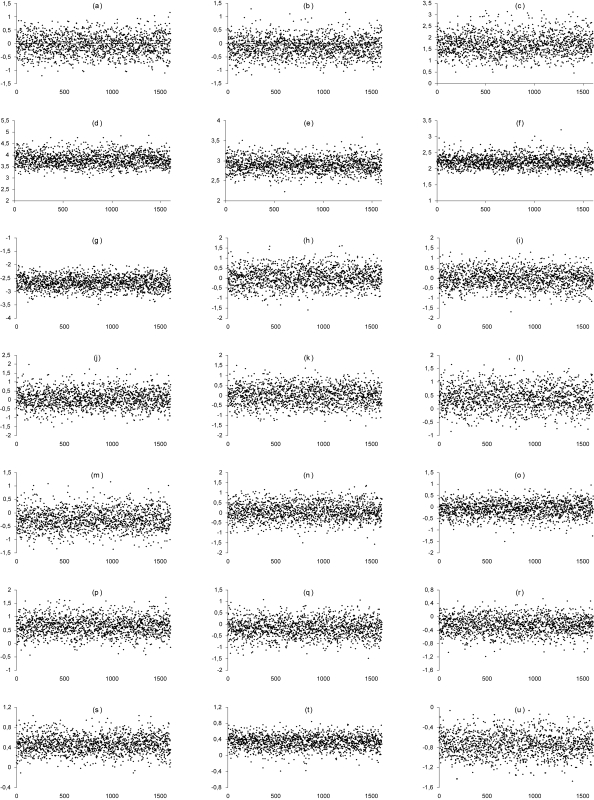
Trace plots of the sample values versus iteration for the parameters *β*_10_ (a), *β*_20_ (b), *β*_30_ (c), *β*_40_ (d), *β*_50_ (e), *β*_60_ (f), *β*_70_ (g), *β*_11_ (h), *β*_21_ (i), *β*_31_ (j), *β*_41_ (k), *β*_51_ (l), *β*_61_ (m), *β*_71_ (n), *β*_12_ (o), *β*_22_ (p), *β*_32_ (q), *β*_42_ (r), *β*_52_ (s), *β*_62_ (t) and *β*_72_ (u).

From the conditional densities for the parameters in *β*, we generated 100,000 Gibbs samples. From this chain, we discarded the first 20,000 (regarded as burn-in samples). The convergence of the Gibbs samples was monitored by standard existing methods [32] and the trace plots obtained are shown in Figure 1. The convergence was observed for all parameters. Prior distributions for the intercept parameters *β*_10_ to *β*_70_ were assumed with fixed hyperparameters based in estimates obtained in the previous analysis without covariates. For example, we noted that the estimated sensitivity of the cervical cytology was 0.536 (see Table 2), and considering the inverse of the logit function, the hyperparameter *a*_10_ is thus given by log (0.536*/*(1 *–* 0.536)). All the other hyperparameter values were chosen to have noninformative priors. Thus, we used an empirical Bayesian modelling approach [33]. For each parameter, we considered every 50th draw, which totalizes a sample of size 1, 600. Considering that a logit link was used, the regression coeffcients in *β* are interpreted as being the logarithm of the odds ratios (OR). These odds ratios represent an association measure between the variables *W*_1_ and *W*_2_ and the operating characteristics of the screening tests.

In Table 6, we have the posterior summaries for the exponential funcion of the parameters of interest in *β*, interpreted as odds ratios. We observe that the 95 percent credible intervals for the parameters *e^β^*^_11_^ to *e^β^*^_71_^ included the value 1, suggesting that there is no evidence for the effect of pregnancy in *e^β^*^_12_^ and *e^β^*^_52_^ *S_e_* and *S_p_* measures for all tests. The parameters e*^β^*^_12_^ and *e^β^*^_52_^ were estimated in 2.033 and 1.615, respectively and its credibility interval does not include the value 1. This result suggests that the sensitivity and the specificity of VIA increases as the age of the women increases. In fact, in the medical literature several authors have described that methods for detection of precursor lesions of cervical cancer have different performances according to the age of women [26, 34, 35]. The prevalence of cervical lesions, as expected, tends to increase as the age of the women increases (*OR* estimated in 0.483 and the respective 95% credible interval do not included the value 1). This effect of age on the prevalence is well-known in the medical literature since the disease is more incident in sexually active women.

## 6. Concluding Remarks

In this article, we introduced a Bayesian approach based on a Markov chain Monte Carlo (MCMC) algorithm that allows the performance measures estimations of diagnostic tests in the presence of covariates and when a gold standard is not available. An advantage of the proposed methodology is the fact that the number of parameters to be estimated is not limited by the number of observations as it happens when we use the method introduced by Hui and Walter [3]. We used the logit link function to relate the covariates linearly to the screening performance measures, but it is possible to use other link functions than the logit function, in according to the nature of the data. For comparison, we also adjusted Bayesian models to estimating the sensitivity and the specificity of the cervical cancer screening tests here presented based on other link functions, as the log-log complementary function, but we do not observe significant changes in the parameter estimates and its inferences (results not shown). However, a mis-specified model could arise from an incorrect link function and the use of model comparison measures, as the Deviance Information Criterion (DIC) of Spiegelhalter et al. [36], allow us to decide which of these functions give us the most appropriate model. An advantage of the logit link function over other functions is that it provides estimates of odds ratios, a meaningful and well-known measure of association.

An important consideration in the use of the proposed model is its dependence on the prior information. In a sensitivity analysis, we noted, for example, that the prevalence of cervical lesions is increased when we used all non-informative prior distributions and other substantial changes in the sensitivity and specificity. In the presented model, the lack of a gold standard is counterbalanced by the introduction of a latent variable *G* (2) that best describe the data simulating a reference test. This latent variable has a Bernoulli distribution with success probability given in function of the performance screening measures and its subjectiveness from the respective prior distributions. Therefore, more accurate results would be given if we incorporate reasonable prior distributions based on prior knowledgement of clinical experts.

Although the proposed model is able to estimate useful performance measures of serial and parallel combinations of the screening tests (see Tables 4 and 5), the relative gains in sensitivity and losses in specificity can be misleading (see details in Franco e Ferenczy [37]). Macaskill et al. [38] argued that the expected number of additional true positive and false positive results (or true negative and false negative results) can be used as the basis for deciding whether to use tests in combination when neither the combined nor a component test shows superior test performance based on their likelihood ratios. Thus, the comparison between the likelihood ratios for two competing tests can be used to assess the incremental gain from an adjunct test. An extension of the presented Bayesian model considering the inclusion of parameters that describe the comparison between likelihood ratios as proposed by Macaskill et al. [38] should be also considered in future studies.

The major shortcoming of the Bayesian estimating method resides in the necessary presumption that the diagnostic tests are statistically and conditionally independent. This presupposition might not be invariably true [39] and alternative methods were proposed by Espeland and Handelman (1989); Yang and Becker (1997) and Dendukuri and Joseph (2001) to address those situations [40, 41, 13]. However, all of these approaches address situations in which the correlation between two screening tests is considered and extensions for three or more tests are not found in the literature. Bayesian models that include the conditional dependence between multiple screening tests should be considered in future studies.

It is also important to point out that the diagnostic tests evaluated in this study have some inherent flaws. The lack of accuracy and reproducibility of cervical cytology is explained by the biological variability, sample quality, subjective interpretation of morphological abnormalities and examiners fatigue derived from repetitive procedures [42]. VIA requires training, although an obvious trend towards examiner subjectivity is always present. HC II results are more reproducible than those of VIA and cervical cytology.

Thus, VIA suggested by Belinson et al. (2001) as a screening method is likely to assume a central role in the prevention of cervical cancer in many countries. This simple and inexpensive method does not require complex technical supplies and it allows diagnosis and treatment at a single visit [25]. Coste et al. (2003) evaluated the performances of conventional cytology, liquid-based cytology and HC II in detecting cervical lesions with a sample of 1,757 women, with a combination of colposcopy and biopsy as the gold standard [43]. This impressive number of colposcopies and biopsies certainly reduced the verification bias, but our results still substantiate the view that this type of statistical modelling could provide reliable results using fewer patients either subjected to the gold standard or simply with no gold standard. This is because of the natural influence of the disease prevalence on the values of PPV and NPV. The prevalence of histologically confirmed cervical abnormalities is necessarily low in a healthy population and screening tests usually have low PPV and high NPV, because true positives are rare and true negatives are abundant [30].

Three Indian studies in the late 1990s provided evidence supporting VIA as a viable alternative to cytology as a primary screening test [44–47]. In one of these studies, Londhe et al. [45] evaluated 12,372 women that underwent VIA, Pap smear and colposcopy in a gynecology outpatient clinic. VIA identified 78 percent of high-grade cervical lesions diagnosed with colposcopy, 3.5 percent more than were identified by cytology. In a 1998 Indian study [46] involving 3,000 women, VIA and cytology (done only by cytotechnicians) performed very similarly (sensitivity ratio of 1.05) in terms of detecting moderate/severe dysplasia. The approximate specificity of VIA in this study was 92.2 percent compared with 91.3 percent for cytology. In a third Indian study published in 1999, Sankaranarayan et al. found that VIA detected significantly more moderate/severe lesions than cytology but its specificity was significantly lower [47]. A large-scale study (over 10,000 women) in Zimbabwe compared the performance of VIA and the Pap smear in the hands of nurse midwives in primary health clinics. Phase II of this study was the first to provide direct estimates of sensitivity/specificity because all women testing negative or positive were offered the reference standard (colposcopy and biopsy, if indicated). In this study, the sensitivity of VIA (for high-grade positivity) was 1.75 times higher than that of cytology (76.7 vs. 44.3 percent respectively) whereas the specificity was 1.4 times lower [26].

The studies mentioned above yielded valuable information regarding the performances of VIA and cytology. Our Bayesian estimates provided performance values that can be compared with the results obtained in the standard manner, that is, with the use of a gold standard. In our estimates, VIA and cytology had similar sensitivity and specificity, as well as PPV and NPV. These figures contradict the previously published direct estimates that reported a superiority of VIA in detecting cervical lesions [46,47]. These differences may be attributable to methodological incompatibilities in sample collection or processing and, as mentioned before, to the inherent diff iculties derived from the variability of cytology and VIA interpretation.

The performance of HPV test in screening settings has been extensively studied, but to a lesser extent in comparison with VIA and cytology. Denny et al. (2000) published their data on 2,944 women subjected to VIA, cytology and HPV testing. In this study, VIA and HPV (>1 RLU) were similar to cytology in their performance of detecting high-grade lesions, but VIA yielded the largest number of false-positives among the three testing modalities [48]. More recently, these same authors [49] published a methodologically similar study testing cervicography, VIA, HPV test and cytology. In this study, 2,754 previously unscreened South African women were subjected to the four exams and VIA detected significantly more high-grade lesions as compared to the other screening tests.

The shortcomings of the tests (as reproducibility, subjective interpretation of results and required training of professional) did not hamper the Bayesian estimates but, in contrast, would enhance the search for realistic estimating equations. Thus, the inclusion of covariates (or control variables) should be encouraged in studies designed for the evaluation of estimating methodologies. According to Parmigiani (2002), prediction models used to support the clinical and health policy decision making need to consider the course of the disease over an extended period of time and draw evidence from a broad knowledge base, including epidemiological cohort and case control studies, randomized clinical trials, expert opinions and more [50]. In these cases, Bayesian decision theory and the tools typically used to describe the uncertainties involved could be extremely useful. The age of the patient is an important covariate in the study of cervical carcinoma precursor lesions. Koss [51] and Schiffman et al. [27,44] did not recommend the use of HPV testing in young women because the prevalence of the virus is exceptionally high in this group and the majority of such infections will spontaneously regress in the short term. Cervical lesions only develop in the presence of persistent HPV infections, thus HPV testing in young women will reveal an excessive number of HPV positive subjects that will never develop HPV-related cervical precancer lesions.

We can conclude that the estimated performances of VIA, HC II and cytology clearly show that the Bayesian method is a remarkable tool for validating diagnostic tests when a gold standard is available for a very limited number of cases or not available at all.

## Figures and Tables

**Table 1:** Results of cervical cytology, VIA and HC II in 809 women who underwent all three tests.

	Cervical cytology +		Cervical cytology −		
	HC II +	HC II −	HC II +	HC II −	Total
VIA+	9	2	15	35	61
VIA−	21	19	87	621	748
Total	30	21	102	656	809

**Abbreviations:** VIA: visual inspection with acetic acid; HC II: Hybrid Capture II.

**Table 2:** Bayesian estimates for sensitivities, specificities, positive and negative predictive values for each screening test, and for the prevalence of cervical lesions.

Test		%	95% CI
Cervical cytology	sensitivity	53.6	42.1–65.0
	specificity	97.0	95.5–98.4
	PPV	55.3	37.4–73.6
	NPV	96.8	94.8–98.3
Visual inspection with acetic-acid	sensitivity	52.9	43.5–62.5
	specificity	93.0	91.0–94.7
	PPV	34.0	22.4–46.2
	NPV	96.6	94.6–98.1
Hybrid Capture II	sensitivity	90.3	76.2–98.7
	specificity	88.7	85.9–91.4
	PPV	35.3	22.8–48.8
	NPV	99.2	97.8–99.9
prevalence		6.4	3.9–9.3

**Abbreviations:** CI: credible interval; PPV: positive predictive value; NPV: negative predictive value.

**Table 3:** Estimates for the number of true positives for each combination of the screening methods and their expected positive predictive values.

Cervical cytology	Test VIA	HC II	Total of sampled individuals	Predicted number of positives	Expected positive predictive value (%)
+	+	+	9	8.87	98.6
+	+	−	2	0.93	46.7
+	−	+	21	17.33	82.5
+	−	−	19	1.37	7.2
−	+	+	15	9.85	65.6
−	+	−	35	0.97	2.8
−	−	+	87	10.39	11.9
−	−	−	621	1.19	0.2

**Abbreviations:** VIA: visual inspection with acetic acid; HC II: Hybrid Capture II.

**Table 4:** Bayesian estimates of the sensitivities and specificities for the combinations between screening tests in serial mode*.

Combination between tests		%	95% CI
Cervical cytology + and VIA +	sensitivity	78.0	70.3–84.8
	specificity	90.2	87.9–92.4
Cervical cytology + and CH II +	sensitivity	95.5	88.7–99.4
	specificity	86.1	82.9–89.1
VIA + and CH II +	sensitivity	95.4	88.5–99.4
	specificity	82.5	79.3–85.6
All tests positive	sensitivity	97.9	94.5–99.7
	specificity	80.1	76.6–83.4

* Positive when all tests were positive and negative otherwise.

**Abbreviations:** VIA: visual inspection of the cervix with acetic-acid; HC II: Hybrid Capture II; CI: credible interval.

**Table 5:** Bayesian estimates of the sensitivities and specificities for the combinations between screening tests in parallel mode*.

Combination between tests		%	95%CI
Cervical cytology + or VIA +	sensitivity	28.3	20.5–36.9
	specificity	99.8	99.7–99.9
Cervical cytology + or CH II +	sensitivity	48.3	36.4–60.3
	specificity	99.7	99.4–99.8
VIA + or CH II +	sensitivity	47.7	36.8–58.2
	specificity	99.2	98.8–99.4
At least one test positive	sensitivity	25.6	17.8–34.2
	specificity	99.98	99.96–99.99

* Positive when at least one of the tests was positive and negative otherwise.

**Abbreviations:** VIA: visual inspection of the cervix with acetic-acid; HC II: Hybrid Capture II; CI: credible interval.

**Table 6:** Posterior odds ratios as association measures between pregnancy and age and the performance measures of the cervical cytology, VIA and HC II.

parameter	measure	mean	SD	95% CI	
*e^β^*^11^	effect of pregnancy on *S_e_*_1_	1.142	0.566	0.420	2.496
*e^β^*^21^	effect of pregnancy on *S_e_*_2_	1.079	0.502	0.400	2.291
*e^β^*^31^	effect of pregnancy on *S_e_*_3_	1.214	0.667	0.413	2.864
*e^β^*^41^	effect of pregnancy on *S_p_*_1_	1.105	0.528	0.416	2.384
*e^β^*^51^	effect of pregnancy on *S_p_*_2_	1.626	0.710	0.670	3.425
*e^β^*^61^	effect of pregnancy on *S_p_*_3_	0.840	0.343	0.381	1.692
*e^β^*^71^	effect of pregnancy on *P*	1.168	0.509	0.479	2.451
*e^β^*^12^	effect of age on *S_e_*_1_	0.973	0.318	0.481	1.694
*e^β^*^22^	effect of age on *S_e_*_2_	2.033	0.699	1.013	3.737
*e^β^*^32^	effect of age on *S_e_*_3_	0.914	0.364	0.393	1.856
*e^β^*^42^	effect of age on *S_p_*_1_	0.804	0.202	0.469	1.239
*e^β^*^52^	effect of age on *S_p_*_2_	1.615	0.282	1.131	2.272
*e^β^*^62^	effect of age on *S_p_*_3_	1.422	0.238	0.996	1.925
*e^β^*^72^	effect of age on *P*	0.483	0.109	0.313	0.738

**Abbreviations:** SD: standard deviation; CI: credibility interval.
